# Editorial: Opioid overdose and harm reduction approaches to combat the looming crisis

**DOI:** 10.3389/fpsyt.2023.1197388

**Published:** 2023-04-20

**Authors:** Sasidhar Gunturu, Sanobar Jaka

**Affiliations:** ^1^Department of Psychiatry, BronxCare Health System, The Bronx, NY, United States; ^2^Department of Psychiatry, Icahn School of Medicine at Mount Sinai, New York City, NY, United States; ^3^Nursing Home Division, Essen Health Care, The Bronx, NY, United States; ^4^Special Care Center, BronxCare Health System, The Bronx, NY, United States

**Keywords:** opioid use disorder, medication-assisted treatment (MAT), cognitive-behavioral therapy (CBT), cannabidiol, Suboxone

Opioid dependence and overdose have rapidly become a major public health crisis in recent years, leading to countless deaths and hospitalizations across the United States. The current opioid epidemic is driven by both the misuse and abuse of prescription opioids, as well as the growing use of heroin and synthetic opioids like fentanyl (1). In the United States alone, more than 106,000 people died from drug overdoses in 2021, with opioids being the leading cause of those deaths (1). Harm reduction approaches, such as medication-assisted treatment (MAT) and distribution of naloxone kits, have been shown to reduce mortality rates in individuals with opioid use disorder (OUD) (2). However, while these approaches can save lives, the risk of relapse and potential overdose remains high (1).

Therefore, there is a pressing need for effective adjunctive treatments to complement MAT and reduce the risks associated with OUD. One promising adjunctive therapy is cannabidiol (CBD), which has been recently studied to understand its role as a substitute in opioid users (3). In addition, cognitive-behavioral therapy (CBT) has been shown to be effective in reducing the risk of relapse by helping individuals to develop coping skills and address underlying psychological issues (4). Four articles in this issue examine the potential of cannabis and CBD as adjunctive treatments, the history and controversies of Suboxone, and the role of cognition in opioid users.

The articles by Sivils et al., and Gharahi et al., provide important insights into different aspects of the opioid epidemic and potential solutions. Harm reduction approaches, like the use of buprenorphine, have shown promise in reducing the negative consequences of opioid use. Additionally, the focus on cognitive function and potential cognitive therapies for individuals with OUD highlights the need for a multifaceted approach to tackling the crisis.

Sivils et al.'s article, “*Suboxone: History, controversy, and open questions*,” discusses the medication buprenorphine and its most popular variation–Suboxone. Buprenorphine is a partial opioid agonist that has been shown to be effective in treating opioid use disorder, with reduced potential for overdose compared to full agonists like methadone. However, Suboxone has also been a subject of controversy with concerns about its diversion and the role of non-selective opioid antagonist naloxone in the medication. This review provides a comprehensive look at the history and current state of buprenorphine and Suboxone, which will be valuable to healthcare providers, policymakers, and individuals affected by opioid use disorder (Sivils et al.).

The article, by Gharahi et al., “*Cognitive network reconstruction in individuals who use opioids compared to those who do not: Topological analysis of cognitive function through graph model and centrality measures*,” focuses on the cognitive dysfunction related to opioid use disorder. The study investigated the interconnected network of cognitive domains in individuals who use opioids compared to controls using graph model analysis. The results show that individuals who use opioids have impaired divided attention, which is a central cognitive function in both groups. The findings suggest that divided attention and its subscales may be a promising target for cognitive therapies and rehabilitation for individuals with opioid use disorder (Gharai et al.).

Suziki et al., report a pilot study investigating the impact of a single dose of CBD on reward- and stress-related neurocognitive processes implicated in relapse among individuals with OUD. The study found that CBD may have promise as an adjunct to medication treatment for OUD by attenuating the brain response to drug-related cues, which in turn may reduce the risk of relapse and overdoses. However, further research is needed to validate these findings and evaluate the potential for CBD as n adjunctive therapy in OUD (Suziki et al.).

The article by Naji et al., reports their prospective cohort study to identify predictors of relapse amongst patients receiving buprenorphine-naloxone therapy, including the association between cannabis use and opioid relapse. They identified factors associated with higher relapse rates, including intravenous drug use, higher doses of buprenorphine-naloxone, and < 2 years in treatment. The study did not find cannabis use to be protective. These findings highlight the need for closer patient follow-up and more stringent treatment protocols to mitigate the risk of relapse and potential overdose. In conclusion, both articles provide valuable insights into the potential of adjunctive treatments for OUD and the factors associated with poor treatment outcomes (Naji et al.).

As we continue to combat the opioid crisis, harm reduction approaches including medication-assisted treatment and adjunctive therapies remain crucial tools to save lives and improve wellbeing for individuals with OUD. FDA's recent approval for over-the-counter sale of Narcan nasal spray is a result of tiring efforts by public health agencies to mitigate this crisis (5). Finding effective adjunctive treatments, such as CBD, and implementing more stringent treatment protocols can help reduce the risk of relapse and potential overdose. By considering a range of approaches, we can work together to combat this crisis and save lives.

## Author contributions

SJ: formulated and edited the manuscript. SG: equally contributed in drafting and editing the editorial. Both authors contributed to the article and approved the submitted version.
